# The Generation of Indole-2,3-quinodimethanes from the Deamination of 1,2,3,4-Tetrahydropyrrolo[3,4-*b*]indoles

**DOI:** 10.3390/molecules25020261

**Published:** 2020-01-09

**Authors:** Alison Rinderspacher, Gordon W. Gribble

**Affiliations:** Department of Chemistry, Dartmouth College, Hanover, NH 03755, USA; alison.rinderspacher@gmail.com

**Keywords:** indole, indole-2,3-quinodimethane, 1,2,3,4-tetrahydropyrrolo[3,4-*b*]indole, deamination, Angeli’s salt

## Abstract

A novel generation of indole-2,3-quinodimethanes via the deamination of 1,2,3,4-tetrahydropyrrolo[s3,4-*b*]indoles is reported.

## 1. Introduction

As an offshoot of the venerable *o*-quinodimethanes [1,2,3,4,5], indole-2,3-quinodimethanes have risen to a position of importance in indole chemistry and they have a fascinating history of exploration and synthetic applications [6,7,8,9,10,11,12,13,14,15].

**Figure 1:**
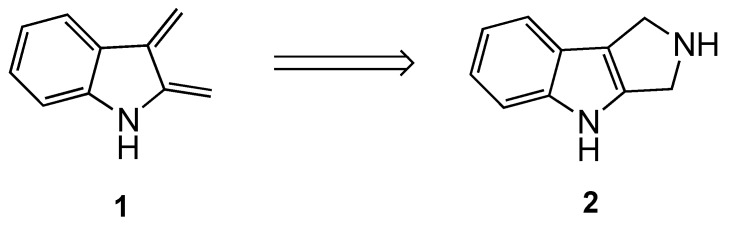
Retrosynthesis of indole-2,3-quinodimethane (**1**) from 1,2,3,4-tetrahydropyrrolo[3,4-*b*]indole (**2**).

In continuation of our synthesis and chemistry of pyrrolo[3,4-*b*]indoles [16,17,18,19,20,21,22], we now report the first generation of an indole-2,3-quinodimethane (**1**) species from the pyrrolo[3,4-*b*]indole (**2**) ring system (Figure 1). Given the availability of pyrrolo[3,4-*b*]indoles and, in particular, 1,2,3,4-tetrahydropyrrolo[3,4-*b*]indoles (i.e., **2**), this possible pathway to the indole-2,3-quinodimethane ring system appeared to be novel and attractive.

**Scheme 1:**
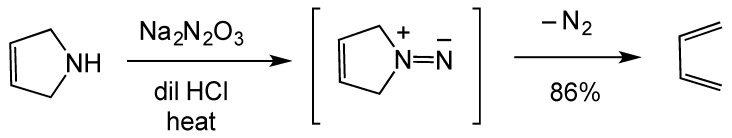
Deamination of 3-pyrrolines with Angeli’s salt.

Moreover, the early work of Lemal and McGregor on the generation of dienes via the stereospecific deamination of 3-pyrrolines with Angeli’s salt (Scheme 1) proffered the feasibility of our concept [23,24].

**Scheme 2:**
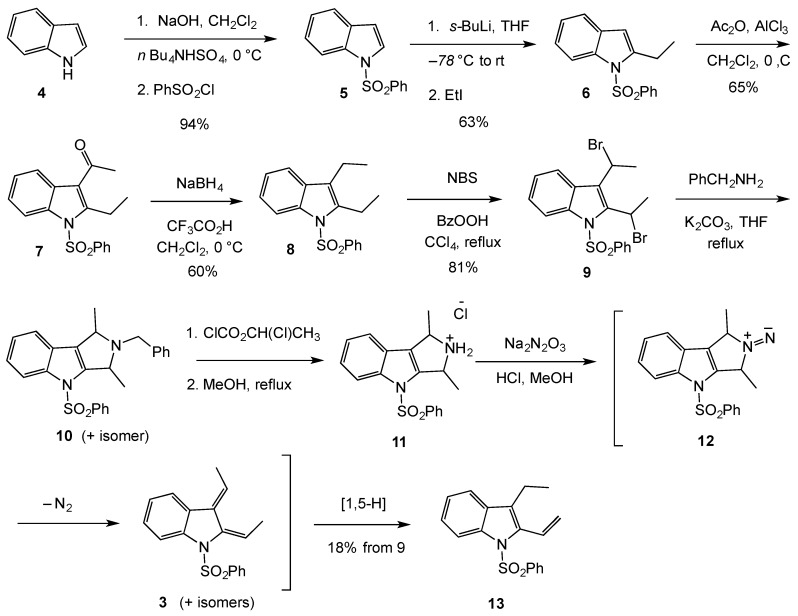
Deamination of 1,2,3,4-tetrahydropyrrolo[3,4-*b*]indole (**11**) leading to 3-ethyl-1-(phenylsulfonyl)-2-vinylindole (**13**).

We initially focused on the generation of 2,3-diethylidene-1-(phenylsulfonyl)indoline **3** from 2,3-diethyl-1-(phenylsulfonyl)indole (**8**) (Scheme 2). However, our first synthesis of **8** from 2-iodoaniline via a Larock indolization [25] with 3-hexyne, followed by indole *N*-phenylsulfonylation proceeded poorly (not shown). Therefore, we adopted the successful route shown in Scheme 2. Indole (**4**) was converted to 1-(phenylsulfonyl)indole (**5**) in standard fashion [26]. Treatment of **5** with *sec*-BuLi (or LDA) and quenching with iodoethane gave 2-ethyl-1-(phenylsulfonyl)indole (**6**) in 63% yield. Exposure of **6** to acetic anhydride and AlCl_3_ in dichloromethane afforded 3-acetyl-2-ethyl-1-(phenylsulfonyl)indole (**7**) in 65% yield [27]. The structure of **7** was confirmed by X-ray crystallography. Interestingly, the reverse sequence of initial C-3 acetylation/reduction followed by C-2 lithiation to give **7** failed, probably due to steric hindrance imparted by the C-3 ethyl group. This was verified by lithiation of 3-ethyl-1-(phenylsulfonyl)indole and quenching with iodomethane to give only traces of 3-ethyl-2-methyl-1-(phenylsulfonyl)indole. Reduction of **7** with NaBH_4_/CF_3_CO_2_H generated 2,3-diethyl-1-(phenylsulfonyl)indole (**8**) in 60% yield. On occasion we found that this reduction stopped at the alcohol stage, the product of which upon treatment with base led to loss of acetaldehyde giving 2-ethyl-1-(phenylsulfonyl)indole. This novel retro-aldol-type reaction was discovered by us many years ago [28]. Indole **8** was treated with *N*-bromosuccinimide and benzoyl peroxide to give the desired dibromoindole **9** in high yield. In a two-step sequence **9** was converted to 1,2,3,4-tetrahydropyrrolo[3,4-*b*]indole **10** with benzylamine, and then debenzylated using Yang’s method [29] to give the hydrochloride salt **11**. Treatment of **11** with Angeli’s salt (Na_2_N_2_O_3_) in the presence of dilute acid gave 3-ethyl-1-(phenylsulfonyl)-2-vinylindole (**13**) via diazine **12** and deamination to generate 2,3-diethylidene **3** via a [1,5-H] shift in 18% overall yield from **9**.

**Scheme 3:**
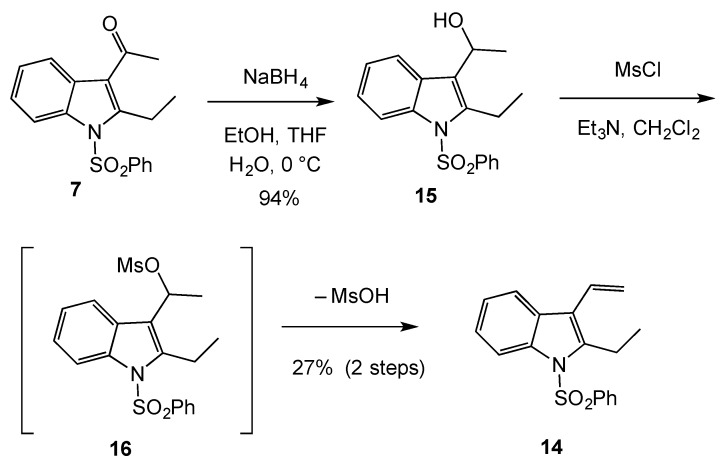
Synthesis of 2-ethyl-1-(phenylsulfonyl)-3-vinylindole (**14**).

The structure of **13** as opposed to the isomeric 2-ethyl-1-(phenylsulfonyl)-3-vinylindole (**14**) was established by both 2D NMR and an independent synthesis of **14** (Scheme 3). In the latter event, reduction of 3-acetylindole **7** afforded alcohol **15**, which was converted to mesylate **16** using Bassoli’s procedure [30]. During the reaction **16** eliminated methanesulfonate to give 2-ethyl-1-(phenylsulfonyl)-3-vinylindole (**14**) in modest yield. The NMR data (COSY, NOESY, chemical shifts) clearly distinguished 3-vinylindole **14** from 2-vinylindole **13**. This observation of a [1,5-H] shift (**3** to **13**) during the formation of an indole-2,3-quinodiethane has been reported by Pindur [31] in his study of 1,4-dimethylpyrano[3,4-*b*]indol-3-one, and by Bergman [32] and Kano [33] during their syntheses of ellipticine.

**Scheme 4:**
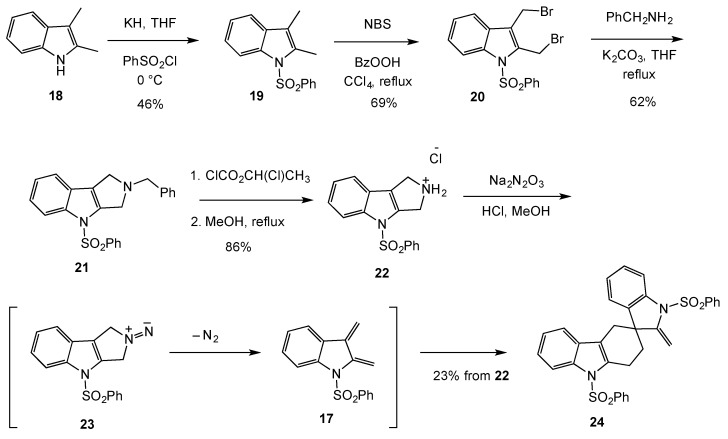
Deamination of pyrrolo[3,4-*b*]indole **22** leading to 2,3-dihydro-2,3-dimethylene-1-(phenylsulfonyl)indole (**17**) and dimer **24**.

To preclude the [1,5-H] shift process, we turned our attention to the possible generation of indole-2,3-quinodimethane **17** as shown in Scheme 4. *N*-phenylsulfonylation of 2,3-dimethylindole (**18**) gave **19**, which was brominated as per Srinivasan [34,35] to give dibromide **20**. Treatment of **20** with benzylamine afforded tetrahydropyrrole **21** in 62% yield. Debenzylation [29] with α-chloroethyl chloroformate gave the hydrochloride salt **22** in 86% yield. Treatment of **22** with Angeli’s salt in the presence of dilute HCl in aqueous MeOH generated indole-2,3-quinodimethane **17** via diazine **23**, and **17** dimerzied to the dimer **24** in 23% yield from **22**. A similar dimerization of an indole-2,3-quinodimethane was described by Martinelli who isolated the BOC-protected version of **24** [6].

**Scheme 5:**
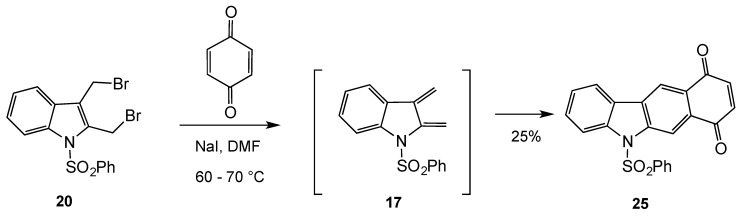
Synthesis of *p*-benzoquinone indole-2,3-quinodimethane **17** adduct **25**.

Attempts to trap **17** with *p*-benzoquinone and dimethyl acetylenedicarboxylate have been unsuccessful. For identification with the anticipated adduct of *p*-benzoquinone with **17**, we prepared adduct **25** following Cava’s conditions [36] (Scheme 5).

The lack of our attempts thus far to trap indole-2,3-quinodimethane **17** with external dienophiles is puzzling, but the isolation of both 2-vinylindole **13** and dimer **24** we believe is clear and convincing evidence of the novel generation of the respective indole-2,3-quinodimethanes **3** and **17** from 1,2,3,4-tetrahydropyrrolo[3,4-*b*]indoles.

## 2. Experimental

### 2.1. General Methods

^1^H-NMR and ^13^C-NMR spectra were recorded on a Varian Unity Plus 300 or 500 T NMR spectrometer. Chemical shifts are reported in parts per million using the residual proton or carbon signal (CDCl_3_: δ_H_ 7.27, δ_C_ 77.23) as an internal reference. The apparent multiplicity (s = singlet, d = doublet, t = triplet, q = quartet, m = multiplet), and coupling constants (in Hz) are reported in that order in parentheses after the chemical shift. All melting points were determined on a Mel-Temp Laboratory Devices apparatus and are reported uncorrected. Thin-layer chromatography (TLC) was performed on Whatman^®^ regular TLC plates. Developed TLC plates were visualized using a 254-nm UV lamp and/or iodine. Flash chromatography employed 230–400 mesh Silicycle^®^ silica gel. Infrared spectra (IR) were recorded with a Perkin Elmer^®^ Series 1600 Fourier transform IR and are reported in reciprocal centimeters. IR spectra were obtained either by dissolving the sample in tetrachloroethene (TCE) or by making salt plates with potassium bromide (KBr). Ultraviolet (UV) spectra were recorded on an Agilent Technologies 8453 UV spectrophotometer. Gas chromatography (GC) and mass spectra (MS) were performed on a Shimadzu GCMS-QP5000 gas chromatograph mass spectrometer. Elemental analyses were performed by Atlantic Microlab Inc. (Norcross, GA, USA). X-ray crystallography was performed by Professor Jerry Jasinski at Keene State College, Keene, NH, USA. High-resolution mass spectrometry was done at the University of Illinois (Urbana-Champaign). Tetrahydrofuran (THF) was dried on a commercial silica gel drying column before use. All alkyllithium reagents were purchased from either Aldrich^®^, Acros^®^, or Alfa Aesar^®^ and were titrated when necessary with diphenylacetic acid. Temperatures of −78 °C were achieved by using an acetone/dry ice bath. Temperatures of −10 °C were reached by employing a sodium chloride solution/ice bath. All reaction flasks were oven-dried at 140 °C. Inert atmospheres of nitrogen were pre-dried by flow through a column of Drierite 4 mesh with indicator. All reactions were done under a positive flow of nitrogen unless otherwise stated. Degassing was accomplished with a manifold using the freeze, pump, and thaw method with nitrogen.

### 2.2. 1-(Phenylsulfonyl)indole (***5***)

A mixture of indole (**4**) (11.8 g, 0.101 mol), sodium hydroxide (12.9 g, 0.323 mol), and tetra-*n*-butylammonium hydrogen sulfate (1.30 g, 0.00383 mol) was stirred in methylene chloride (100 mL) at 0 °C. A solution of benzenesulfonyl chloride (15.5 mL, 0.122 mol) in methylene chloride (40 mL) was added dropwise via addition funnel. Upon addition of the solution the color of the suspension turned from grayish to blue to dark red. After 40 min it had turned light orange and it was allowed to warm to room temperature. The reaction mixture was filtered through a Celite pad and the solvent was evaporated in vacuo, leaving a red oil behind. The oil was treated with methanol (40 mL) and was subsequently stored at 4 °C. Crystal formation was observed almost instantaneously. Pale peach-colored crystals of **5** were obtained after vacuum filtration (24.4 g, 0.0948 mol, 94%): mp 75.5–77 °C (lit. [37] mp 77.5–79 °C); ^1^H-NMR (CDCl_3_) δ 8.01 (d, 1H, *J* = 8.4 Hz), 7.89 (d, 2H, *J* = 7.2 Hz), 7.58 (d, 1H, *J* = 3.6 Hz), 7.53 (d, 2H, *J* = 5.7 Hz), 7.45 (t, 2H, *J* = 7.8 Hz), 7.32 (t, 1H, *J* = 6.9 Hz), 7.23 (d, 1H, *J* = 5.7 Hz), 6.68 (d, 1H, *J* = 3.6 Hz); UV λ_max_ (95% EtOH) 212, 250, 283 nm.

### 2.3. 2-Ethyl-1-(phenylsulfonyl)indole (***6***)

*sec*-Butyllithium in hexane (1.4 M, 4.10 mL, 5.74 mmol) was added dropwise via syringe to a solution of 1-(phenylsulfonyl)indole (5) (1.22 g, 4.75 mmol) in anhydrous THF (30 mL) at −78 °C. The resulting bright orange solution was stirred for 30 min, after which iodoethane (0.38 mL, 4.75 mmol) was added quickly via syringe. The dark orange-brown solution was allowed to warm to room temperature and was subsequently poured onto saturated aqueous ammonium chloride (50 mL). The organic layer was extracted with diethyl ether (3 × 25 mL), washed with brine (2 × 25 mL), and dried with anhydrous magnesium sulfate. The solvent was evaporated in vacuo to give clear, colorless crystals of 6 (0.858 g, 3.00 mmol, 63%): mp 97–97.5 °C (lit. [38] mp 96–97.5 °C); ^1^H-NMR (CDCl_3_) δ 8.19 (d, 1H, *J* = 8.4 Hz), 7.75 (d, 2H, *J* = 8.4 Hz), 7.53 (t, 1H, *J* = 8.7 Hz), 7.45–7.39 (m, 3H), 7.30–7.20 (m, 2H), 6.41 (s, 1H), 3.04 (q, 2H, *J* = 7.2 Hz), 1.36 (t, 3H, *J* = 7.2 Hz).

### 2.4. 3-Acetyl-2-ethyl-1-(phenylsulfonyl)indole (***7***)

Aluminum chloride (0.57 g, 4.3 mmol) and acetic anhydride (0.20 mL, 2.1 mmol) were stirred in methylene chloride (20 mL) at 0 °C. After 20 min a solution of 2-ethyl-1-(phenylsulfonyl)indole (6) (0.19 g, 0.67 mmol) in methylene chloride (3.5 mL) was added dropwise to the yellow suspension via addition funnel. The solution was stirred for 2.5 h at 0 °C, after which it was poured over crushed ice (~100 mL). The organic layer was extracted with methylene chloride (3 × 20 mL), washed with brine (2 × 25 mL) and saturated aqueous sodium bicarbonate (25 mL), and was dried with anhydrous magnesium sulfate. The solvent was evaporated on the rotary evaporator, yielding a brown oil, which was stored at 4 °C. White crystals of 7 were obtained after vacuum filtration (0.14 g, 0.44 mmol, 65%): mp 71–73 °C (lit. [39] mp 92–93 °C); ^1^H-NMR (CDCl_3_) δ 8.30–8.28 (m, 1H), 7.90–7.88 (m, 1H), 7.81 (d, 2H, *J* = 8.5 Hz), 7.59 (t, 1H, *J* = 7.5 Hz), 7.47 (t, 2H, *J* = 7.5 Hz), 7.37–7.33 (m, 2H), 3.38 (q, 2H, *J* = 7.5 Hz), 2.66 (s, 3H), 1.37 (t, 3H, *J* = 7.5 Hz); ^13^C-NMR (CDCl_3_) δ 195.9, 149.7, 139.2, 136.3, 134.5, 129.8 (2C), 127.4, 126.7 (2C), 125.0, 124.7, 121.0, 120.5, 115.2, 32.3, 21.1, 15.6; IR ν(TCE) 3394, 2978, 2471, 1884, 1801, 1672, 1533, 1448, 1385, 1224, 1182, 1122, 1093, 1056 cm^−1^.

### 2.5. 2,3-Diethyl-1-(phenylsulfonyl)indole (***8***)

A sodium borohydride pellet (1.1 g, 29 mmol) was partially dissolved in trifluoroacetic acid (25 mL) at 0 °C under nitrogen. A solution of 3-acetyl-2-ethyl-1-(phenylsulfonyl)indole (7) (0.52 g, 1.6 mmol) in methylene chloride (30 mL) was added dropwise via addition funnel. The yellow solution was stirred overnight. It was diluted with distilled water (75 mL) and basified with sodium hydroxide pellets. The organic layer was extracted with methylene chloride (3 × 50 mL), washed with brine (3 × 50 mL), and was dried with anhydrous magnesium sulfate. The solvent was evaporated on the rotary evaporator, yielding white crystals with a tiny amount of red-brown oil. The product was purified via column chromatography (2:1 hexanes:ethyl acetate) to give 8 as a yellowish, crystalline substance (0.30 g, 0.96 mmol, 60%): mp 87–88 °C; ^1^H-NMR (CDCl_3_) δ 8.19 (d, 1H, *J* = 7.5 Hz), 7.68 (d, 2H, *J* = 7.5 Hz), 7.49 (t, 1H, *J* = 6.5 Hz), 7.42 (d, 1H, *J* = 7.0 Hz), 7.38 (t, 2H, *J* = 8.0 Hz), 7.28–7.22 (m, 2H), 3.01 (q, 2H, *J* = 7.0 Hz), 2.63 (q, 2H, *J* = 7.5 Hz), 1.31 (t, 3H, *J* = 7.0 Hz), 1.17 (t, 3H, *J* = 7.5 Hz); ^13^C-NMR (CDCl_3_) δ 139.3, 138.8, 137.0, 133.6, 130.9, 129.3 (2C), 126.4 (2 C), 124.2, 123.6, 123.0, 118.7, 115.5, 20.0, 17.7, 16.0, 15.0; MS *m*/*z* 313 (M), 172 (100%), 157, 144, 115, 77; HRMS Calcd for C_18_H_19_NO_2_S: 313.1136; Found: 313.1138; Anal. Calcd for C_18_H_19_NO_2_S: C, 68.98; H, 6.11; N, 4.47; S, 10.23. Found: C, 69.22; H, 6.06; N, 4.48; S, 10.07.

### 2.6. 2,3-Bis-(1-bromoethyl)-1-(phenylsulfonyl)indole (***9***)

2,3-Diethyl-1-(phenylsulfonyl)indole (**8**) (0.477 g, 1.52 mmol) was stirred in carbon tetrachloride (20 mL). *N*-Bromosuccinimide (0.548 g, 3.08 mmol) and benzoyl peroxide (0.0535 g, 0.387 mmol) were added. The orange suspension was heated to reflux for 2.5 h, after which it was allowed to cool to room temperature. The light orange-brown reaction mixture was poured onto distilled water (50 mL). The organic layer was extracted with ethyl acetate (3 × 25 mL), washed with distilled water (2 × 25 mL), and dried with anhydrous magnesium sulfate. The solvent was evaporated in vacuo, yielding **9** as a red-brown oil (0.579 g, 1.23 mmol, 81%); MS *m*/*z* 471 (M), 166, 115, 84 (100%); HRMS Calcd for C_18_H_17_Br_2_NO_2_S: 468.9347; Found: 468.9357.

### 2.7. 2-Benzyl-1,3-dimethyl-4-(phenylsulfonyl)-1,2,3,8-tetrahydropyrrolo[3,4-b]indole (***10***)

A solution of benzylamine (0.16 mL, 1.5 mmol) in anhydrous THF (20 mL) was added slowly via addition funnel to a refluxing orange suspension of 2,3-bis-(1-bromoethyl)-1-(phenylsulfonyl)indole (**9**) (0.58 g, 1.2 mmol) and potassium carbonate (0.58 g, 4.2 mmol) in anhydrous THF (30 mL). It was refluxed for 14 h after which it was allowed to cool to room temperature. The light orange suspension was filtered through a Celite pad with methylene chloride. The solvent was evaporated in vacuo yielding **10** as an orange oil, which was immediately taken on to the next step without further purification: MS *m*/*z* 311 (M − 105), 170 (100%), 142, 115, 106, 91, 77.

### 2.8. 1,3-Dimethyl-4-(phenylsulfonyl)-1,2,3,4-tetrahydropyrrolo[3,4-b]indole Hydrogen Chloride (***11***)

A solution of α-chloroethyl chloroformate (0.17 mL, 1.5 mmol) in anhydrous methylene chloride (20 mL) was added dropwise via addition funnel to the reaction mixture of crude 2-benzyl-1,3-dimethyl-4-(phenylsulfonyl)-1,2,3,8-tetrahydropyrrolo[3,4-*b*]indole (**10**) (0.51 g, 1.2 mmol) in anhydrous methylene chloride (30 mL), with stirring at −10 °C. The reaction mixture turned orange-brown. After 35 min the solvent was removed via vacuum pump. Methanol (20 mL) was added to the orange-brown residue. The reaction mixture was refluxed for 55 min, after which it was allowed to cool to room temperature. The solvent was evaporated in vacuo yielding **11** as a brown oil with a white solid. This crude product was taken on to the next step without any further purification: MS *m*/*z* 363 (M), 220 (100%), 141, 114, 77.

### 2.9. 3-Ethyl-1-(phenylsulfonyl)-2-vinylindole (***13***)

The crude product **11** (0.45 g, 1.2 mmol) was stirred in a mixture of methanol (20 mL) and distilled water (20 mL) at room temperature. Angeli’s salt (0.12 g, 1.2 mmol) was added quickly to the latté-colored reaction mixture. Hydrochloric acid (0.6 M, 2.1 mL, 1.2 mmol) was added via syringe. The reaction mixture was stirred for 10 min at room temperature, after which it was refluxed for 90 min and allowed to cool to room temperature, then cooled to −78 °C, and allowed to gradually warm to room temperature. Methylene chloride (20 mL) was added to the yellow-brown reaction mixture and the layers were separated. The aqueous layer was extracted with methylene chloride (3 × 20 mL) and the combined organic extracts were washed with distilled water (2 × 30 mL), and dried with anhydrous magnesium sulfate. The solvent was evaporated in vacuo to afford a red-brown oil, which was purified via column chromatography, using first hexanes and then methylene chloride as eluent. The final fractions of the first column were purified via column chromatography, employing first *n*-pentane and then methylene chloride as eluent. Indole **13** was obtained as an orange oil (0.069 g, 0.22 mmol, 18%, 3 steps): ^1^H-NMR (CDCl_3_) δ 8.21 (d, 1H, *J* = 8.4 Hz), 7.88–7.84 (m, 1H), 7.73 (d, 1H, *J* = 9.6 Hz), 7.55–7.41 (m, 4H), 7.35 (t, 3H, *J* = 8.1 Hz), 7.28 (t, 3H, *J* = 8.4 Hz), 7.13 (q, 1H, *J*_X_ = 11.4 Hz), 5.60 (dd, 1H, *J*_M_ = 11.4 Hz), 5.41 (dd, 1H, *J*_A_ = 17.4 Hz), 2.73 (q, 2H, *J* = 7.5 Hz), 1.17 (t, 3H, *J* = 7.5 Hz); ^13^C-NMR (CDCl_3_) δ 133.7, 129.0 (3C), 128.0, 127.2, 126.9 (3C), 125.2, 123.9, 119.5, 115.5, 18.2, 15.1; MS *m*/*z* 311 (M), 170, 154, 144, 115, 84 (100%), 77, 51; HRMS calcd for C_18_H_17_O_2_NS: 311.0980; Found: 311.0971.

### 2.10. 2-Ethyl-1-(phenylsulfonyl)indol-3-yl-ethanol (***15***)

Ethanol (15 mL) and distilled water (15 mL) were added to a solution of 3-acetyl-2-ethyl-1-(phenylsulfonyl)indole (**7**) (0.107 g, 0.325 mmol) in THF (10 mL), and stirred at room temperature. The light-orange reaction mixture was cooled to 0 °C. Sodium borohydride powder (0.0272 g, 0.719 mmol) was added quickly. The pale yellow reaction mixture was stirred overnight and then poured onto brine (100 mL). The organic layer was extracted with methylene chloride (3 × 25 mL), washed with brine (2 × 40 mL), and dried with anhydrous magnesium sulfate. The solvent was evaporated in vacuo to give **15** as a pale yellow oil (0.100 g, 0.305 mmol, 94%): ^1^H-NMR (CDCl_3_) δ 8.21 (d, 1H, *J* = 8.5 Hz), 7.81 (d, 1H, *J* = 8.0 Hz), 7.72 (d, 2H, *J* = 7.0 Hz), 7.49 (t, 1H, *J* = 8.0 Hz), 7.38 (t, 2H, *J* = 7.5 Hz), 7.28 (t, 1H, *J* = 11.0 Hz), 7.23 (t, 1H, *J* = 8.0 Hz), 5.17 (q, 1H, *J* = 6.5 Hz), 3.16–3.09 (m, 1H), 3.03–2.95 (m, 1H), 1.58 (d, 3H, *J* = 6.5 Hz), 1.29 (t, 3H, *J* = 7.5 Hz); ^13^C-NMR (CDCl_3_) δ 139.2, 138.7, 137.0, 133.8, 129.3 (2C), 128.4, 126.3 (2C), 124.3, 123.6, 120.7, 115.2, 64.2, 23.7, 19.9, 16.1; IR ν(TCE) 3383, 3069, 2976, 2933, 2876, 1761, 1604, 1449, 1376, 1226, 1177, 1092, 1053, 957, 776, 752, 686, 584; MS *m*/*z* 311 (M − 18), 170 (100%), 155, 143, 128, 115, 77.

### 2.11. 2-Ethyl-1-(phenylsulfonyl)-3-vinylindole (***14***)

Methanesulfonyl chloride (0.03 mL, 0.4 mmol) and triethylamine (0.5 mL, 4 mmol) were added via syringe to a solution of 2-ethyl-1-(phenylsulfonyl)-indol-3-yl-ethanol (**15**) (0.1 g, 0.3 mmol) in anhydrous methylene chloride (15 mL), while stirring at room temperature. The solution was stirred for 7 days after which methylene chloride (25 mL) and saturated aqueous ammonium chloride (30 mL) were added. The organic layer was extracted with methylene chloride (3 × 25 mL), washed with saturated aqueous sodium bicarbonate (30 mL) and distilled water (30 mL), and dried with anhydrous magnesium sulfate. The solvent was evaporated in vacuo affording a yellow oil which was purified via column chromatography (first 2:1 hexanes: ethyl acetate, then methylene chloride). Indole **14** was obtained as a yellow oil (0.03 g, 0.09 mmol, 27%, 2 steps): ^1^H-NMR (CDCl_3_) δ 8.23 (d, 1H, *J* = 7.5 Hz), 7.73 (d, 3H, *J* = 7.5 Hz), 752 (t, 1H, *J* = 7.5 Hz), 7.40 (t, 2H, *J* = 7.5 Hz), 7.33–7.26 (m, 2H), 6.76 (q, 1H, *J*_X_ = 11.5 Hz), 5.75 (dd, 1H, *J*_M_ = 17.5 Hz), 5.45 (dd, 1H, *J*_A_ = 11.5 Hz), 3.11 (q, 2H, *J* = 7.5 Hz), 1.32 (t, 3H, *J* = 7.5 Hz); ^13^C-NMR (CDCl_3_) δ 133.8 (2C), 129.4 (3C), 127.7, 126.4 (3C), 124.5 (2C), 124.0, 119.9, 116.9, 115.2 (2C), 20.1, 15.5; HRMS calcd for C_18_H_17_O_2_NS: 311.0980; Found: 311.0977.

### 2.12. 2,3-Dimethyl-1-(phenylsulfonyl)indole (***19***)

Potassium hydride (2.62 g of 30 wt% mineral oil dispersion, 65.2 mmol) was washed with hexanes (1 × 20 mL, 2 × 10 mL). The remaining hexanes were removed in vacuo. Anhydrous THF (40 mL) was added via cannula. A solution of 2,3-dimethylindole (**18**) (2.01 g, 13.8 mmol) in anhydrous THF (20 mL) was added via cannula to the white suspension. Benzenesulfonyl chloride (1.77 mL, 13.8 mmol) was added dropwise via syringe to the yellow-brown reaction mixture. The reaction mixture turned green. It was poured onto crushed ice (250 mL). The organic layer was extracted with diethyl ether (3 × 60 mL), washed with brine (2 × 60 mL), and dried with anhydrous magnesium sulfate. The solvent was evaporated in vacuo yielding **19** as white-brown crystals, which were washed with methanol to give white crystals (1.81 g, 6.34 mmol, 46%): mp 131–133 °C (lit. [35] mp 140 °C); ^1^H-NMR (CDCl_3_) δ 8.20 (d, 1h, *J* = 6.9 Hz), 7.75 (d, 1H, *J* = 7.5 Hz), 7.52 (t, 1H, *J* = 7.2 Hz), 7.43–7.38 (m, 3H), 7.31–7.24 (m, 3H), 2.53 (s, 3H), 2.14 (s, 3H).

### 2.13. 2,3-Bis(bromomethyl)-1-(phenylsulfonyl)indole (***20***)

2,3-Dimethyl-1-(phenylsulfonyl)indole (**19**) (1.03 g, 3.61 mmol), *N*-bromosuccinimide (NBS) (1.33 g, 7.39 mmol), and benzoyl peroxide (0.12 g, 0.87 mmol) were stirred in carbon tetrachloride (47 mL). The yellow reaction mixture was heated to reflux and refluxed for 3.5 h, after which it was allowed to cool to room temperature. The succinimide was filtered off via gravity filtration and then through a cotton plug. Distilled water was added (50 mL). The organic layer was extracted with methylene chloride (3 × 50 mL), washed with brine (2 × 50 mL), and dried with anhydrous magnesium sulfate. The solvent was evaporated *in vacuo* to give **20** as yellow crystals, which were recrystallized with 4:1 hexanes:methylene chloride (1.11 g, 2.50 mmol, 69%): mp 132–133 °C (lit. [35] mp 138 °C); ^1^H-NMR (CDCl_3_) δ 8.12 (d, 2H, *J* = 7.8 Hz), 7.96 (d, 2H, *J* = 7.5 Hz), 7.64–7.35 (m, 5H), 5.15 (s, 2H), 4.67 (s, 2H).

### 2.14. 2-Benzyl-4-(phenylsulfonyl)-1,2,3,4-tetrahydropyrrolo[3,4-b]indole (***21***)

A solution of benzylamine (0.09 mL, 0.8 mmol) in anhydrous THF (20 mL) was added dropwise via addition funnel to a refluxing brown suspension of 2,3-dibromomethyl-1-phenylsulfonyl)indole (**20**) (0.31 g, 0.60 mmol) and potassium carbonate (0.35 g, 2.5 mmol) in anhydrous THF (30 mL). After 15 h the yellow-brown suspension was allowed to cool to room temperature and was filtered through a Celite pad with ethyl acetate. The solvent was evaporated in vacuo, yielding an orange oil, which was purified via column chromatography (2:1 hexanes: ethyl acetate). Indole **21** was obtained as a crystalline solid (0.14 g, 0.37 mmol, 62%): mp 149–151 °C (lit. [20] mp 151–152 °C); ^1^H-NMR (CDCl_3_) δ 8.00 (d, 1H, *J* = 7.8 Hz), 7.85 (d, 2H, *J* = 7.2 Hz), 7.56 (t, 1H, *J* = 7.2 Hz), 7.44 (q, 5H, *J* = 7.8 Hz), 7.39 (s, 1H), 7.36–7.31 (m, 2H), 7.24–7.18 (m, 2H), 4.31 (t, 2H, *J* = 3.6 Hz), 4.05 (s, 2H), 3.95 (t, 2H, *J* = 3.6 Hz).

### 2.15. 4-(Phenylsulfonyl)-1,2,3,4-tetrahydropyrrolo[3,4-b]índole hydrogen chloride (***22***)

A clear, colorless solution of α-chloroethyl chloroformate (0.07 mL, 0.6 mmol) in anhydrous methylene chloride (10 mL) was added dropwise via addition funnel to a pale orange solution of 2-benzyl-4-(phenylsulfonyl)-1,2,3,4-tetrahydropyrrolo[3,4-*b*]indole (**21**) (0.2 g, 0.5 mmol) in anhydrous methylene chloride (10 mL) with stirring at −10 °C. The peach-colored solution was stirred for 1 h. The solvent was removed via vacuum pump. Methanol (10 mL) was added to the peach-colored, solid residue. The white suspension was refluxed for 1.75 h. The reaction mixture was allowed to cool to room temperature. The solvent was evaporated in vacuo, yielding **22** as a gray-white solid. The solid was recrystallized with a mixture of hexanes:methylene chloride (1:1). A white solid was obtained (0.1 g, 0.4 mmol, 86%): mp 219–220°; ^1^H-NMR (CDCl_3_) δ 7.98 (d, 1H, *J* = 9.3 Hz), 7.91 (d, 2H, *J* = 7.2 Hz), 7.59 (d, 1H, *J* = 6.9 Hz), 7.52 (t, 2H, *J* = 7.8 Hz), 7.41–7.27 (m, 3H), 4.99 (s, 2H), 4.69 (s, 2H); Anal. Calcd for C_16_H_15_ClN_2_O_2_S: C, 57.40; H, 4.52; Cl, 10.59; N, 8.37; S, 9.58. Found: C, 55.58; H, 4.50; Cl, 10.58; N, 7.79; S, 8.96.

### 2.16. 2′-Methylene-1′,9-bis(phenylsulfonyl)-1,2,4,9-tetrahydrospiro[carbazole-3,3′-indoline; (Dimer ***24***)

4-(Phenylsulfonyl)-1,2,3,4-tetrahydropyrrolo[3,4-*b*]índole hydrogen chloride (**22**) (0.371 g, 1.11 mmol) and Angeli’s salt (0.112 g, 1.13 mmol) were stirred in a 1:1 mixture of methanol (15 mL) and distilled water (15 mL). Hydrochloric acid (0.6 M, 1.85 mL, 1.11 mmol) was added via syringe. The reaction mixture was stirred at room temperature for 27 min, after which it was refluxed for 2 h. It was allowed to cool to room temperature and was subsequently was cooled to −78 °C. It was allowed to gradually warm up to room temperature. The organic layer was extracted with methylene chloride (3 × 25 mL), washed with saturated aqueous sodium bicarbonate (2 × 25 mL), and dried with anhydrous magnesium sulfate. The solvent was evaporated *in vacuo*, yielding a red-brown oil, which was purified via column chromatography (2:1 hexanes: ethyl acetate). Dimer **24** was obtained as a tan solid (0.0731 g, 0.129 mmol, 23%): mp 134.5–135 °C; ^1^H-NMR (CD_2_Cl_2_) δ 7.97 (d, 1H, *J* = 7.5 Hz), 7.91 (t, 1H, *J* = 7.8 Hz), 7.84 (d, 2H, *J* = 7.8 Hz), 7.63–7.53 (m, 2H), 7.51–7.43 (m, 4H), 7.37 (t, 3H, *J* = 8.4 Hz), 7.31 (d, 2H, *J* = 7.2 Hz), 7.24 (t, 3H, *J* = 7.5 Hz), 5.83–4.78 (m, 2H), 4.27 (s, 2H, *J* = 3.6 Hz), 4.04 (s, 2H), 3.92 (s, 2H, *J* = 3.6 Hz); ^13^C-NMR (CDCl_3_) δ 134.7 (2C), 134.6 (2C), 129.9 (4C), 127.0 (4C), 125.5 (2C), 125.4 (2C), 124.5 (2C), 123.9 (2C), 119.9 (2C), 119.7 (2C), 114.6 (2C), 114.5 (2C), 52.3, 51.0, 49.2, 47.6; MS *m*/*z* 566 (M), 532, 457, 387, 315, 284, 245, 220, 173, 142, 91 (100%); HRMS Calcd for C_32_H_26_N_2_O_4_S_2_: 566.1334; Found: 566.1328.

### 2.17. 5-Phenylsulfonyl-5H-benzo[b]carbazole-7,10-dione (***25***)

2,3-Dibromomethyl-1-(phenylsulfonyl)indole (**20**) (0.50 g, 1.1 mmol), sodium iodide (1.2 g, 8.0 mmol), and *p*-benzoquinone (0.26 g, 2.4 mmol) were stirred in DMF (10.0 mL). The dark red-brown reaction mixture was heated to 78 °C and later cooled to 73.5 °C. It was heated for 18 h and was subsequently allowed to cool to room temperature. The dark-brown mixture was filtered through a Celite pad with methylene chloride. The filtrate was poured onto aqueous sodium bisulfite. The organic layer was extracted with methylene chloride (3 × 20 mL), washed with brine (2 × 20 mL), and dried first with sodium sulfate, then with anhydrous magnesium sulfate. The solvent was evaporated in vacuo giving a brown oil, which was purified via column chromatography. Quinone **25** was obtained as a bright yellow solid (0.11 g, 0.28 mmol, 25%): mp 214 °C dec.; ^1^H-NMR (CDCl_3_) δ 9.00 (s, 1H), 8.66 (s, 1H), 8.40 (d, 1H, *J* = 8.7 Hz), 8.08 (d, 1H, *J* = 7.8 Hz), 7.93 (d, 2H, *J* = 7.5 Hz), 7.67 (t, 1H, *J* = 7.5 Hz), 7.55–7.46 (m, 2H), 7.40 (t, 2H, *J* = 8.7 Hz), 7.05 (q, 2H, *J*_AB_ = 10.2 Hz); ^13^C-NMR (CDCl_3_) δ 184.9, 184.7, 139.5, 139.1, 134.7 (2C), 130.0 (2C), 129.7 (2C), 128.3, 126.9 (2C), 125.2, 125.1 (2C), 121.7 (2C), 119.4, 115.3, 113.4 (2C); UV λ_max_ (95% EtOH) 195, 216, 284 nm; MS *m*/*z* 387 (M), 246 (100%), 218, 190, 164, 141, 115, 77; HRMS Calcd for C_22_H_13_NO_4_S: 387.0565; Found: 387.0556.

### 2.18. Angeli’s Salt (Sodium Trioxodinitrate)

The procedure of Addison was used [40]. A 21% solution of sodium ethoxide (17.7 mL, 47.4 mmol) in ethanol was added dropwise at 0 °C to a saturated solution of hydroxylamine hydrochloride (1.10 g, 15.8 mmol) in ethanol (30mL). A light-orange suspension formed. After 80 min the resulting precipitate was filtered off via gravity filtration. *n*-Butyl nitrate (2.32 mL, 15.8 mmol) was added via syringe at room temperature to the yellow filtrate. The filtrate was cooled in an ice bath. A cream-colored solid (0.764 g, 7.71 mmol, 49%) was collected via vacuum filtration and immediately used for the deamination reaction step.
